# Experimental Study on the Dissolution Characteristics and Microstructure of Carbonate Rocks under the Action of Thermal–Hydraulic–Chemical Coupling

**DOI:** 10.3390/ma16051828

**Published:** 2023-02-23

**Authors:** Jinzhu Meng, Sili Chen, Junxiang Wang, Zhi Chen, Jingyu Zhang

**Affiliations:** School of Architecture and Civil Engineering, Shenyang University of Technology, Shenyang 110870, China

**Keywords:** carbonate rock, thermal–hydraulic–chemical coupling, dissolution effect, computed tomography scanning, microstructure

## Abstract

Microdamage in a rock induces a change in the rock’s internal structure, affecting the stability and strength of the rock mass. To determine the influence of dissolution on the pore structure of rocks, the latest continuous flow microreaction technology was used, and a rock hydrodynamic pressure dissolution test device simulating multifactor coupling conditions was independently developed. The micromorphology characteristics of carbonate rock samples before and after dissolution were explored using computed tomography (CT) scanning. To conduct the dissolution test on 64 rock samples under 16 groups of working conditions, 4 rock samples under 4 groups were scanned by CT under working conditions, twice before and after corrosion. Subsequently, the changes in the dissolution effect and pore structure before and after dissolution were quantitatively compared and analyzed. The results show that the dissolution results were directly proportional to the flow rate, temperature, dissolution time, and hydrodynamic pressure. However, the dissolution results were inversely proportional to the pH value. The characterization of the pore structure changes before and after sample erosion is challenging. After erosion, the porosity, pore volume, and aperture of rock samples increased; however, the number of pores decreased. Under acidic conditions near the surface, carbonate rock microstructure changes can directly reflect structural failure characteristics. Consequently, heterogeneity, the presence of unstable minerals, and a large initial pore size result in the formation of large pores and a new pore system. This research provides the foundation and assistance for predicting the dissolution effect and evolution law of dissolved pores in carbonate rocks under multifactor coupling, offering a crucial guide for engineering design and construction in karst areas.

## 1. Introduction

With the improvement of the social economy, some of the subterranean projects are starting to gain traction. Karst gradients, foundation instability, karst water flow, and karst collapse have a detrimental impact on the foundations of structures and pose a considerable risk to the safety and financial investment of engineered construction. Moreover, due to the anisotropy of rock, its interior contains holes, cracks, microcracks, and microplanes, and its mesocharacteristics will significantly affect its strength, stability, and seepage characteristics. Hence, to conduct engineering construction in karst areas and to improve the stability of engineering structures and later safety maintenance, the characteristics of the rock dissolution law in soluble rock areas must be understood. Moreover, there should be research on rock pore structure and prediction of its development trends to assure early identification and safety evaluation of karst geological disasters. These are crucial for the safe development of rock mass engineering and are vital for material research.

There have been several karst-related studies, including those on karst landforms [1,2], hydrogeology [3,4], karst environment [5,6], and so on. Since the 1970s, researchers have been studying the experimental dissolution of carbonate rock under temperature, mechanical, and chemical conditions [7]. The research includes carbonate rock dissolution and precipitation [8,9,10,11], dissolution reaction law and dynamic process [12,13], the dissolution mechanism of karst development features [14,15,16], and the dissolution rate of rock samples and their influencing factors [17,18,19,20,21]. The main factors affecting the dissolution effect of carbonate rocks are found to be temperature [22], flow rate [23,24], hydrodynamic pressure [20,25,26,27], and pH value [28]. Based on macromechanism research through dissolution tests, there are numerous test methods for observing the internal mesostructure of materials in the research of the mesocharacteristics of rocks, such as scanning electron microscopy (SEM), nuclear magnetic resonance (NMR), and CT [29,30,31]. NMR and CT can be used to analyze, as well as detect, rock samples of any shape and size with a small number of samples, high speed, a nondestructive process, and high accuracy. NMR can directly detect fluid and its distribution in rock samples. Additionally, the porosity of the rock sample can also be measured more accurately using this technique, including analyzing the size distribution, connectivity, and mobile fluid of the rock. Furthermore, it has obvious advantages in quantitatively characterizing the pore defects in rock, and realizes the quantitative evaluation of the damaged state of rock [32,33]. Zhang et al. [34] revealed the evolution of pore size, pore volume, and permeability during the dolomite reservoir dissolution process. Meanwhile, Xiao et al. [35] show that regardless of whether the pore structure is good or bad, it is better to use the pore composition to evaluate the pore structure. Diffusion-weighted imaging (DWI) has always been of interest to scholars. Fheed proposed using DWI as it can show the arrangement of fissures in rock cores or allow conclusions to be drawn regarding the permeability of a reservoir sample. In order to resolve the specifics of naturally bendy carbonate pore networks consisting of narrow dissolution channels, determining a major fluid flow direction is necessary [36,37,38,39]. Krzyżak et al. proposed a more advanced technique of diffusion tensor imaging (DTI) for characterizing rock core samples. Upon obtaining the diffusion tensor (DT), the parameters of the DT could be observed in both 2D and 3D. Each parameter was explained, and its value in characterizing pore space was examined [40,41]. Meanwhile, Fheed also applied zero echo time (ZTE) imaging using a high-field 9.4 T MRI scanner to study the relationship of the local water saturation level and the sample microstructure. In comparison with high resolution 3D micro-CT images, ZTE seems to be especially suitable for studying porous and fractured carbonate rocks [42]. In comparison, the ray method of CT scanning is ideal for studying the internal structure of minerals and geotechnical materials. This technology was developed in the 1970s and was initially used for nondestructive testing in the medical field [43]. However, in the 1990s, computer science and technology made significant advances, while industrial CT gradually increased. With the rapid development and maturity of technology recently, it has been popularized and applied to geotechnical engineering from medical and industrial fields and gradually applied to civil engineering, water conservancy and hydropower, and other fields as a nondestructive testing technology. Raynaud [44] was the first to use X-ray CT scanning to analyze the scanning sections of rock samples. He discovered that rock volume deformation correlated with changes in ray density. The formation of microcracks accompanies rock salt microdamage, and the micropores in rock salt change accordingly, affecting the change in rock salt density. CT observed structural changes in rock in a nondestructive, real-time, and dynamic manner without requiring a complex sample preparation process or causing structural damage [45]. Berg et al. [46], Wildenschild and Sheppard [47], Blunt et al. [48], and Luquot and Gouze [49] provided a necessary method for studying the internal mesostructural evolution of rock salt during dissolution and for evaluating the changes of secondary pores formed by karst under various conditions to study the pore system in reservoirs. To better understand fluid flow in complex porous media, Garing et al. [50], Bird et al. [51], Bultreys et al. [52], Cnudde and Boone [53], Rabbani et al. [54], Dong et al. [55], Freire-Gormaly et al. [56], and Noiriel et al. [57] extracted three-dimensional pore network information using segmentation, binarization, skeleton, and other methods. They defined it using porosity distribution, pore radius, pore throat radius, pore throat length, and coordination number. Rötting et al. [58] discovered that significant dissolution occurred only under certain conditions and pore sizes. Then, Krebs et al. [59] and Yao et al. [60] used NMRI and microfocused computed tomography (μCT) techniques to image rock samples before and after dissolution. Both realized 3D visualization and fine characterization of rocks using μCT as reference visualization technology since it can measure the scale as well as the relationship between pore and fracture accurately. They further completed the fine description of the scale by including spatial development as well as distribution characteristics of pores and fissures.

Regarding the dissolution mechanism of carbonate rocks, previous studies on rock salt primarily began at the macroscopic level. They explained the microscopic dissolution and the “driving force” of pore development from the perspectives of dissolution kinetics and diagenesis. Many factors were thought to control the change in pore structure caused by dissolution, such as fluid temperature, pressure, pH value, porosity, permeability, and crystal size, but the studies were mostly based on a single environmental equilibrium state, with little research on multifactor control systems in different occurrence environments. Furthermore, the solution preparation of the experiment’s reaction was complicated, the reaction time was long, and the accuracy was low. The acid fluid moved and reacted in the internal pores of the carbonate rocks during diagenesis. However, existing research has contributed enormous knowledge to pore system reform under different pressure and temperature conditions. Fredd and Foggler [61,62,63], Golfier et al. [64], and Tansey and Balhoff [65] evaluated the micro-dissolution process, pore structure property change, and micro-dissolution mechanism under the action of thermal–hydro–chemical coupling. However, they ignored the relationship between rock and dissolution.

Consequently, to investigate the effect of dissolution on the pore structure of carbonate rocks under thermal–hydraulic–chemical coupling conditions, the latest continuous flow microreaction technology was used. Limestone samples from the Dalian coastal area were chosen for the research. Furthermore, an independently developed rock hydrodynamic pressure dissolution test device simulated multifactor coupling conditions. The degree of influence of various influencing factors on rock mass dissolution under thermal–hydraulic–chemical coupling conditions was investigated, and previous test device limitations were overcome. The dissolution rate of carbonate rock and the chemical composition of karst water were quantitatively studied using an orthogonal experimental design, linear regression analysis, and variance analysis on the test results. Hence, the relationship between dissolution rate and influencing factors was quantified, and the results were obtained. Next, CT was used to obtain slice images of the material’s internal structure. The mechanism and distribution law of dissolved pores in rock salt dissolution was studied. The surface morphology of rock samples before and after the dissolution was compared and analyzed microscopically. Next, the characteristics of the microscopic dissolution process and pore changes of carbonate rocks were investigated. The changes in microstructure parameters before and after dissolution were quantitatively analyzed, providing a theoretical basis for the cause, distribution, and prediction of dissolved pores in carbonate rocks.

## 2. Test Equipment and Experimental Methodology

### 2.1. Test Samples

**Figure 1:**
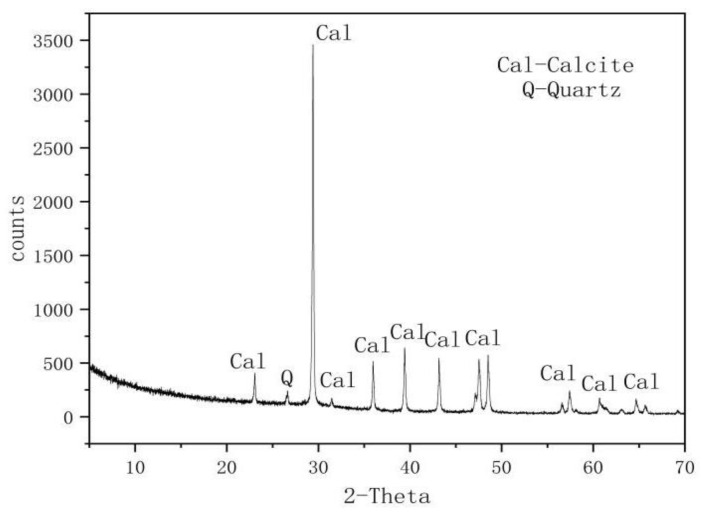
Supplementary figure of the X-ray diffraction whole-rock mineral analysis [7].

The rock samples used in the experiment were gathered from the Dalian Bay shoreline. This region’s karst formations, including karst caves, dissolved gaps, and dissolution funnels, are the result of groundwater and seawater erosion as well as the effect of the surrounding structure. Table 1 comprises a list of the samples’ mineral compositions that were subjected to X-ray diffraction (XRD) analysis, and Figure 1 shows the results of whole-rock XRD mineral analysis. The predominant mineral in the rock samples was calcite, with a small amount of quartz.

Table 2 lists the chemical makeup of samples obtained from X-ray fluorescence spectroscopy. CaO is the main ingredient in the rock samples with 55.41% concentration, indicating that these are carbonate rocks.

**Figure 2:**
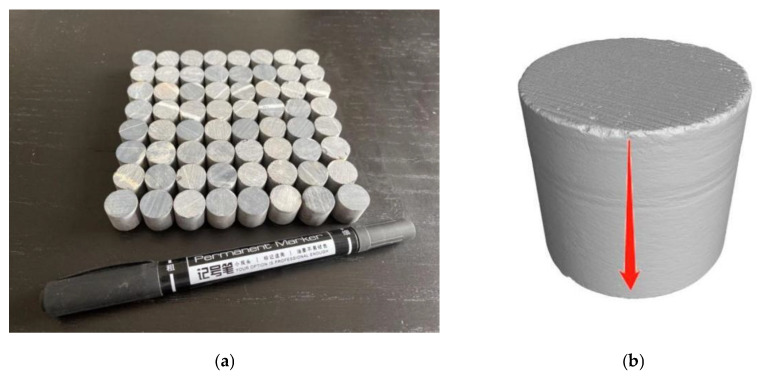
Picture of rock samples: (**a**) rock specimens; (**b**) location map of the rock sample.

Considering the low accuracy of CT scanning of large-sized specimens and the demand of subsequent mechanical research, the commonly used large-sized specimens were changed into Φ 12 mm × 12 mm micro-cylindrical rock samples (Figure 2a) using a drill core machine. The rock samples were washed with deionized water and then dried in a drying box at 105 °C for 12 h. The samples were weighed with an electronic balance using a measuring range of 0–120 g and an accuracy of 0.01 mg. They were numbered, and the weight W1 of the samples was recorded before dissolution. Figure 2b shows the solution’s infiltration direction on the rock salt side.

### 2.2. Test Device

#### 2.2.1. Dissolution Test Equipment

**Figure 3:**
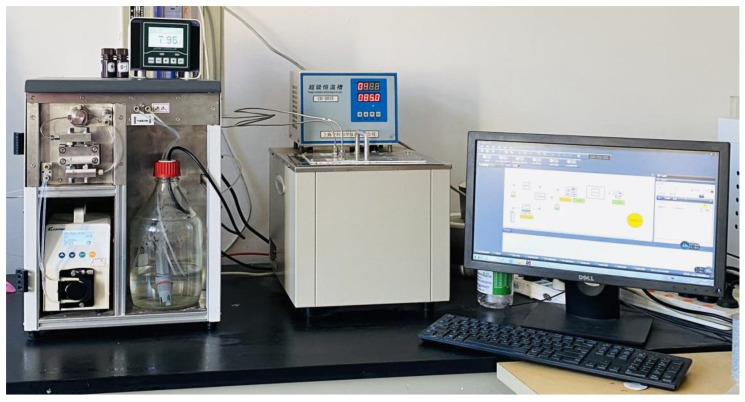
Self-made YDYR-2 rock hydrodynamic pressure dissolution test equipment [7].

A set of YYDR-2 rock hydrodynamic pressure dissolution test equipment (Figure 3) based on microreaction technology was created using the latest continuous flow microreaction technology [7]. The multifactor dynamic simulation corrosion test of carbonate rocks under different hydrochemical conditions, temperature conditions, pressure conditions, and hydrodynamic conditions was realized.

The reaction chamber in the dissolution reaction system can simultaneously hold four cylindrical samples within Φ13 mm × 25 mm. Between the upper cover and the main body is a cover design for an inlet filter seat that forms four umbrella-shaped structures on the upper and lower sides through a three-degree design. Furthermore, an outlet filter seat cover is designed between the main body and the lower cover, and 316L stainless steel filters were installed in the inlet and outlet filter seats to filter liquid impurities and rock residues damaged by dissolution.

#### 2.2.2. CT Scanning Equipment

As a nondestructive testing technology, CT can transmit an object through an X-ray beam and obtain the internal three-dimensional structural characteristics of the object under nondestructive testing conditions, such as the distribution of pores and material composition.

Micronano focus CT 3D scanning technology was used to study the microstructure characterization of rock samples to quantitatively analyze the internal structural characteristics of rock samples before and after dissolution. The diondo d2 high-resolution all-around micronano focus CT detection system was used for CT scanning. For the detection range, the specimen was rotated at 360° and irradiated. The collected 2D projection images were reconstructed using computer data to obtain 3D CT volume data. Subsequently, the data were visually analyzed to form a 3D perspective view.

#### 2.2.3. NMR Equipment

The NMR equipment is the MacroMR12-150H-I MRI Analyzer produced by Numai Electronic Technology Co., Ltd, Shanghai, China. It can realize the simulation study under various conditions. The pore size distribution, porosity, pore connectivity, and some physical properties of rocks can be obtained by measuring the relaxation characteristics of hydrogen protons contained in the fluid of internal rock pores. Then, the internal microstructure of rocks can be analyzed qualitatively or quantitatively. Unfortunately, it cannot achieve DWI and DTI detection and analysis of the related topic.

### 2.3. Test Plan

The test was divided into two parts. The first was a carbonated water dissolution test of limestone. The second was a CT scanning of rock salt samples before and after dissolution to investigate the influence of dissolution on the physical and mechanical properties of rock salt, particularly the changes in the microscopic results of rock salt before and after dissolution.

#### 2.3.1. Orthogonal Dissolution Test Scheme

By using the orthogonal design software Latin, an orthogonal experimental design method, and an orthogonal table the experimental simulation scheme was determined; some representative points are selected from the comprehensive test for experiments based on the orthogonality. These representative points are uniformly dispersed, neat, and comparable. As the main method of analytical factor design, the orthogonal test design is efficient, fast, and economical. It can significantly reduce workload while determining the best combination level for various factors. Five influencing factors were chosen: pH, hydrodynamic pressure, temperature, current speed, and duration, and each was uniformly set at four levels (Table 3).

Using orthogonal table L16(45) for scheme design, 16 groups of simulation test schemes were obtained to carry out the dissolution test of 64 rock samples under 16 groups of working conditions, and the final values of each factor level are shown in Table 4.

#### 2.3.2. CT Scanning Test Scheme

Because of the restrictions of the test setup, rock samples No. 4 (5-4,6-4,7-4,8-4) from groups 5 to 8 of the 16 groups of the dissolution test are chosen. These will be referred to as 5#, 6#, 7#, and 8#, respectively, from now on. Two CT tests before and after dissolution are conducted. The scanning conditions of rock samples are as follows: one layer is scanned every 0.008 mm from top to bottom, and 1400 layers are scanned continuously. The internal mesostructural damage changes and macro-dissolution damage of the specimen at the same position before and after dissolution were compared by measuring the composition characteristics of the internal structure before rock salt dissolution. This was for CT macroscopic image analysis and a thorough understanding of the rock microstructure.

#### 2.3.3. NMR Test Scheme

Four rock samples (5#, 6#, 7#, and 8#) selected in the CT test were tested by NMR, wherein the internal porosity and permeability of the rock samples were quantitatively analyzed. These could then be compared with the CT scanning results.

### 2.4. Test Steps

#### 2.4.1. Initial CT Scans of Carbonate Rocks

Four rock samples (5#–8#) were scanned by CT before dissolution; the CT machine was debugged, the scanning interval was determined, and the number of scanning layers was chosen. The procedure corresponded with that of the first batch of rock salt samples and the test results were recorded and analyzed.

#### 2.4.2. Dissolution of Carbonate Rocks

After the initial CT scan of the four sets of rock samples, they were sequentially placed in the sample bin with the other 12 sets of rock samples in the marked direction of infiltration. The dissolution test was repeated according to the direction below.

**Figure 4:**
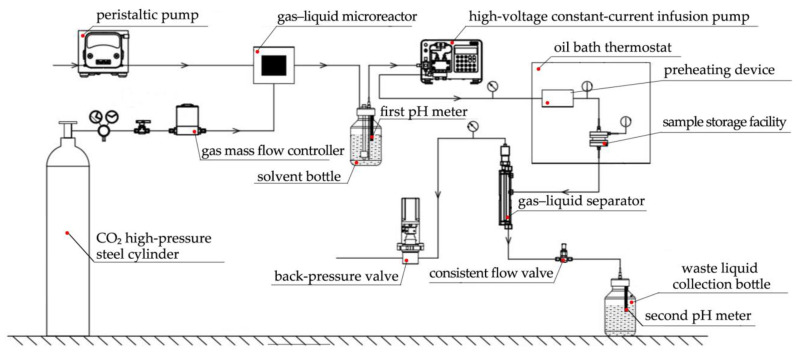
Flow chart of the dissolution test [7].

The CO_2_ gas inlet was set using the gas mass flow controller and the water current speed using the peristaltic pump. At the gas–liquid micro-mixer, the gas–liquid mixing in real time was completed. Using the first pH meter, the solvent’s pH level was continuously monitored in the solvent container. Further, the rock sample was kept in the reactor, and the oil bath thermostat’s temperature was adjusted to the predetermined test temperature. Then, the high-pressure constant-current infusion pump’s flow rate was set in accordance with the demands of the test’s four test circumstances. To create a sample warehouse with a specific pressure and flow rate of carbonic acid water flowing at a fixed temperature, the back pressure valve’s pressure value was gradually modified. Following the reaction, the gas–liquid separator’s separated gas is immediately released through the back pressure valve and into the atmosphere. Through a steady flow valve, the separated liquid is released into the waste liquid collection bottle. The pH of the solution is continuously monitored by the second pH meter in the bottle. After the test, the solution was collected both before and after dissolution in a high-density polyethylene plastic bottle. The rock sample should be taken out of the reactor, rinsed with deionized water, dried, weighed again (W2), and its volume should be calculated (Figure 4).

#### 2.4.3. Second CT Scanning of Carbonate Rocks

Following dissolution, the samples (5#–8#) underwent a second CT scan utilizing the identical procedures as the first, and test results were again documented and examined.

#### 2.4.4. The NMR of Carbonate Rocks

After the second CT scan, NMR tests were performed on the rock samples (5#, 6#, 7#, and 8#), wherein the test data were recorded and analyzed.

## 3. Results and Discussion

### 3.1. Analysis of Dissolution Results and Influencing Factors

The dissolution amount is the weight difference (W1−W2) between the rock sample before and after dissolution. The dissolution amount to mass before the test ratio is the unit mass dissolution amount, and the dissolution rate = dissolution amount (mg)/weight before dissolution (g). Table 5 shows the average dissolution rate of the 16 groups of test conditions.

In working condition 4, the dissolution rate is 364.43 mg/g, while in working condition 16, it is 3.69 mg/g. There are numerous variations in the dissolution rates.

**Figure 5:**
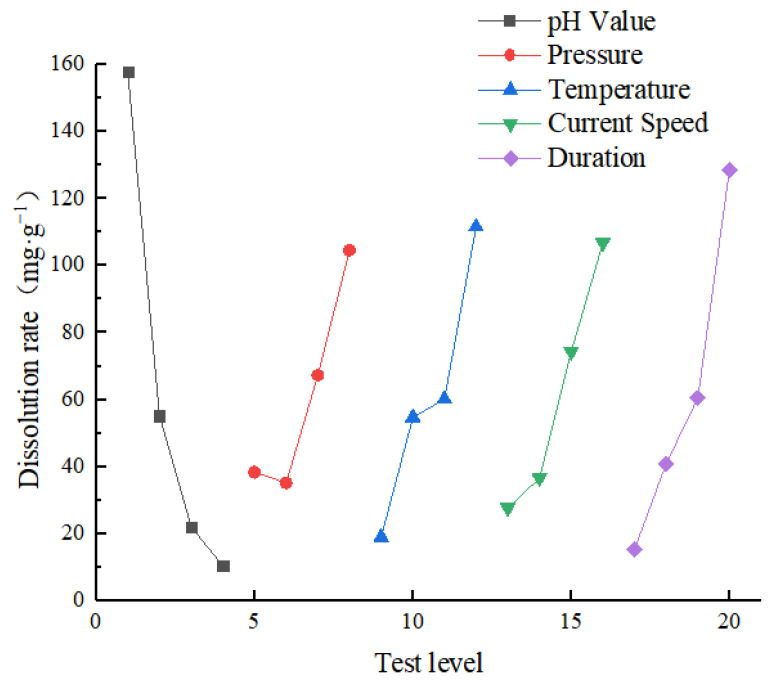
Sensitivity analysis of dissolution rate.

An evaluation of each factor’s primary and secondary influence on the test indexes using range analysis of orthogonal test results shows that the greater the range, the greater the influence of the factor’s level change on the test index. Thus, the range is the most important factor. Table 5 shows the average value and range of each level of each factor. Conversely, Table 6 shows the range analysis results for the dissolution rate. Figure 5 shows the sensitivity analysis chart of the dissolution rate. The graph shows that the influence factor pH is the most significant and that the temperature, current speed, and pressure are all similar. Consequently, the sensitivity of each factor to the dissolution rate was pH > time > temperature > current speed > pressure, implying that pH was an important factor in the dissolution rate of specimens.

Table 7 shows the variance analysis table of the sample dissolution rate. It can be observed from the table that the solution pH had the greatest contribution rate to the sample dissolution rate, which was 42.36%, followed by time, which was 21.37%. Hence, the pH value had a significant impact on the rate of sample dissolution.

### 3.2. Analysis of CT Scan Test Results

To determine the controlling effect of the pore structure on the evolution of corroded pores, the internal pores of the four groups of rock samples were compared and analyzed before and after dissolution. The pore structure characteristics, such as porosity, pore size distribution, pore number, and pore volume, were quantitatively evaluated.

#### 3.2.1. Spatial Distribution Characteristics of Pores

The gray scale is indicated by the light and dark degrees of the CT image, while the change in material density shows the change in the gray value in the image. The bright white area with the larger grayscale is the high-density area, mostly made of rock salt particles and cement. The small gray scale is a low-density area with pores, holes, cracks, and other parts on the sample’s cross-section. CT scanning parameters should be consistent with the cross-section, allowing the comparison of internal mesostructural damage and macro-dissolution damage of rock salt at the same position before and after dissolution. The scanning layer thickness was 0.008 mm in the experiment, and three scanning layers of 150, 750, and 1350 were chosen for comparison. Moreover, the spatial distribution of pores and fractures and the mineral distribution characteristics were qualitatively analyzed.

**Figure 6:**
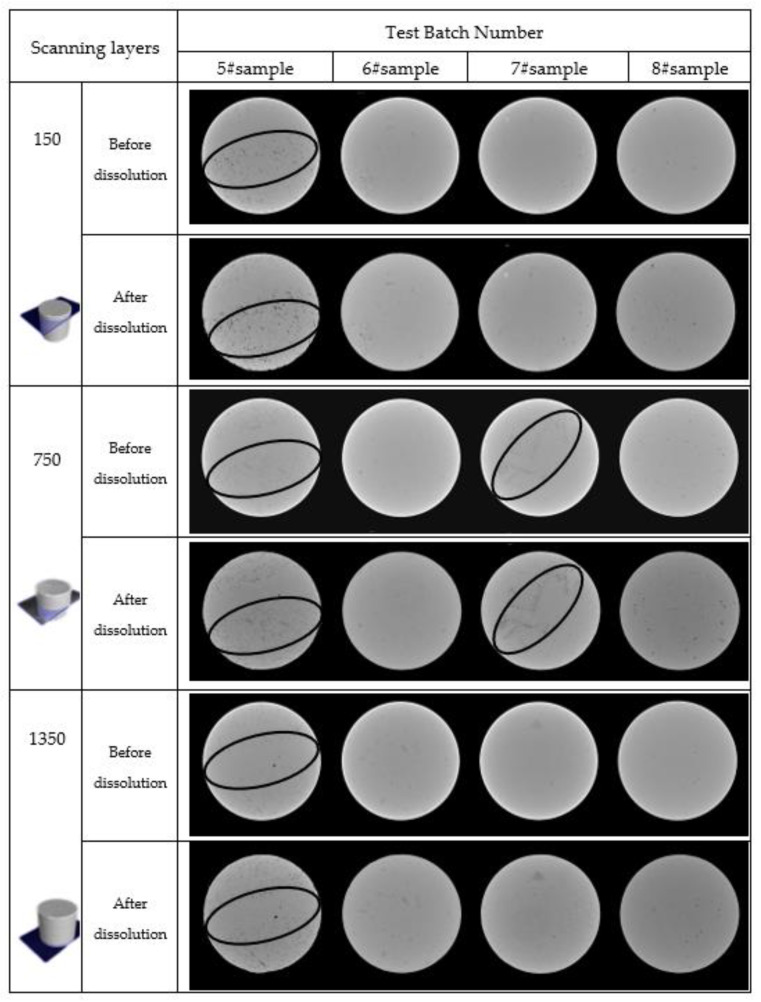
Cross-sectional view of CT scan.

The salt samples’ water inflow and infiltration directions are 150–750–1350 horizons. Figure 6 depicts a cross-sectional view of the CT scanning of the four groups of samples before and after the dissolution of the three horizons. The CT scan images before rock salt dissolution in different zones had different light and dark ranges, indicating that the density distribution was uneven. Furthermore, the rock salt observed was a medium with nonuniform initial damage, primarily micropores and microcracks.

There are many abnormally low-density areas in the three horizons in the initial scanning image of sample 5#. The density of the sample was unevenly distributed, indicating a concentrated area of pores and cracks, which was essentially a microcrack development zone with an extremely fragile structure. After salt dissolution, the abnormally low-density areas grew extensively, a few pores enlarged, and fractures began to penetrate, indicating that the particles, arrangement forms, and fracture structures in the rock salt layers were extremely uneven. The presence of low-density areas and dissolution increased the dispersion of the rock salt’s internal structure, characterized by dissolution. Some rock salt-soluble substances were removed from the solution, allowing fractures to form. One hundred fifty horizons performed well, porosity varied greatly, and the density of different areas increased unevenly in the other horizons.

The gray scale distribution of three horizons in the initial scanning image of rock sample 7# was relatively uniform, with only a few abnormally low-density areas; the closer to the edge of the image before salt dissolution, the greater the grayscale, which means the greater the density of this area. The initial rock salt edge density was high, the density of the central area was low, and the structure was fragile. When the image’s brightness was compared before and after dissolution, the grayscale of the edge area of each horizon increased noticeably. However, the grayscale of the other areas did not. That is, the initial rock salt’s edge density was high, the density of the central area was low, and the structure was fragile. When the image’s brightness was compared before and after dissolution, the grayscale of the edge area of each horizon increased noticeably. In contrast, the grayscale of the other areas did not.

The gray scale distribution of the three horizons was relatively uniform in the initial scanning images of rock samples 6# and 8#, with only a few abnormally low-density areas, scattered pores, and little damage to the internal structure. There was no obvious change in the overall grayscale of the image before and after the dissolution of the rock salt, and only the local area of the rock salt was abnormal. Hence, the grayscale increased.

#### 3.2.2. Quantitative Characterization of Pore Structure

1. Porosity change

**Figure 7:**
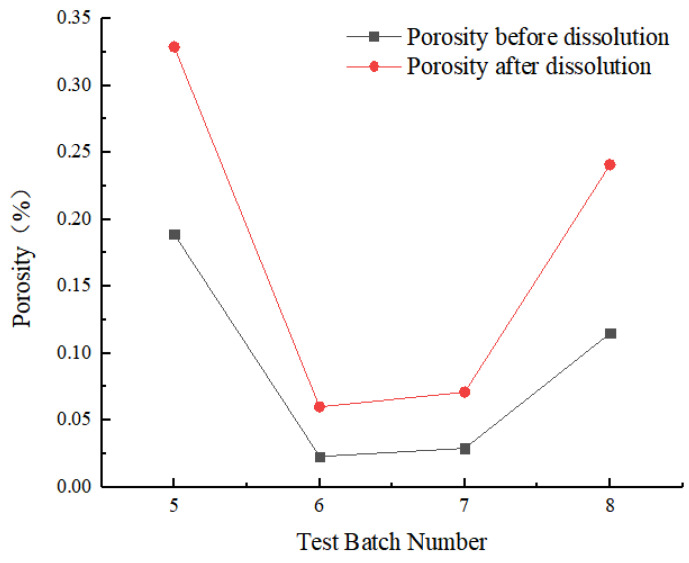
Porosity comparison before and after dissolution.

The ratio of pore volume to total volume obtained from CT scanning images of rock samples, including closed pores and connected pores, was defined as porosity. The initial porosities of the four rock samples were 0.189%, 0.023%, 0.029%, and 0.115%, respectively. Porosities were compared before and after dissolution under four different test conditions. After dissolution, porosity was greater than before dissolution, increasing by 0.140%, 0.038%, 0.042%, and 0.126%, respectively (Figure 7). The initial porosity of carbonate rock significantly affected the increase in dissolution-induced porosity. Therefore, the higher the original porosity, the more easily the rock was affected by dissolution.

**Figure 8:**
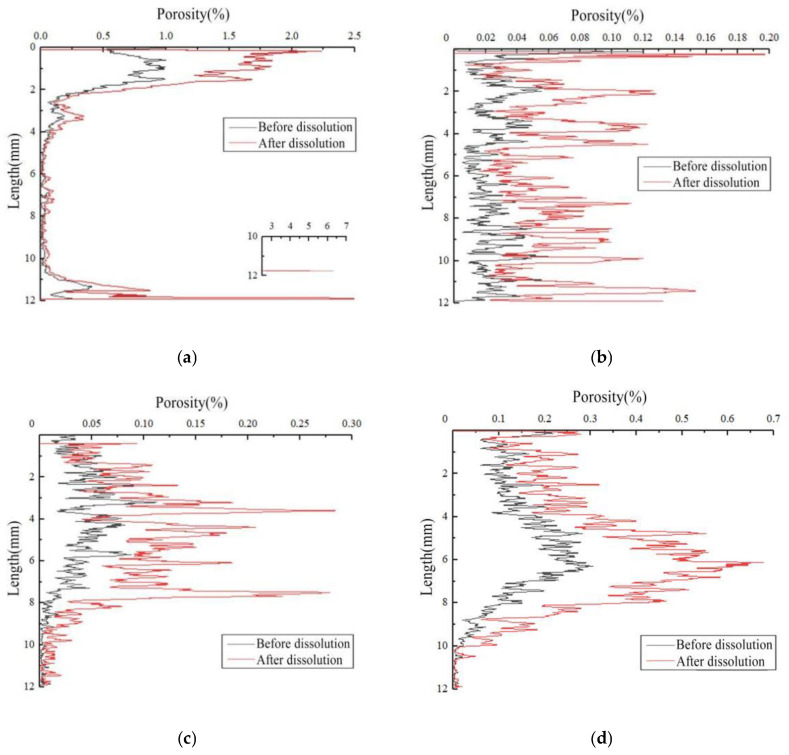
Changes of porosity distribution of carbonate samples before and after dissolution: (**a**) sample 5#, (**b**) sample 6#, (**c**) sample 7#, and (**d**) sample 8#.

Figure 8 shows the change in the porosity distribution of the rock samples before and after dissolution under different test conditions. The initial porosity of the different rock samples (before dissolution) varied due to the heterogeneity of rock samples. The flow of the dissolution liquid was from the top to the bottom. The porosity distribution showed that dissolution could improve overall porosity. Furthermore, the change in porosity ran throughout the entire sample, indicating that the sample corroded in three directions of the rectangular coordinate system. Since the porosity of the upper part was greater than that of the lower part, particularly at the top of the sample, it reacted with the carbonic acid solution entering the reactor, causing it to erode faster than the rest of the rock sample.

2. Pore number and volume change

The number and volume of pores in carbonate samples were determined before and after dissolution under various test conditions. In the four groups of rock samples, the number of pores ranged from 2716 to 19,683. The number of pores decreased by 5429, 95, 331, and 4406, but the pore volume increased by 1.63 mm^3^, 0.53 mm^3^, 0.58 mm^3^, and 1.88 mm^3^, respectively, after dissolution. The reduction in pore number and the increase in pore volume indicated that some pores merged to form larger ones.

**Figure 9:**
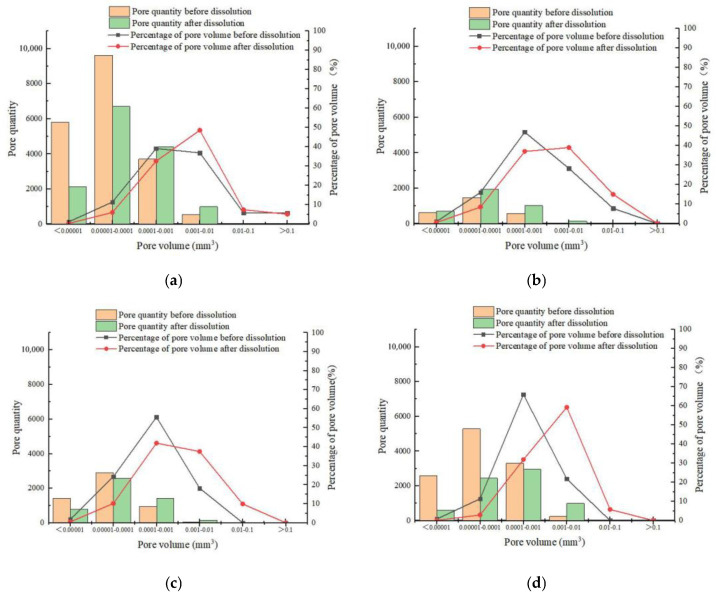
Relationship between pore quantity and the corresponding volume percentage for different pore volumes: (**a**) sample 5#, (**b**) sample 6#, (**c**) sample 7#, and (**d**) sample 8#.

Following the overall characterization of pores, the number and volume percentage of pores were statistically classified from the perspective of single pore volume to deeply analyze the pore structure and distribution of the four groups of the rock samples. Figure 9 shows that as pore volume increased, the number of pores and the percentage of pore volume increased at first and then decreased. Before dissolution, the percentage distribution of pore volume had the characteristics of the right deviation under different test conditions. However, after dissolution, the percentage distribution of pore volume has the characteristics of left deviation, and most of the kurtosis values decreased, indicating that the distribution peak was flatter than before dissolution. When the pore volume of a single pore exceeded 0.0001 mm^3^, the percentage of pore volume increased significantly, reaching the highest values of 48.6%, 37.5%, 41.9%, and 59.3%, and increasing by 11.7%, 10.7%, 19.3%, and 37.5%, respectively, exceeding that before dissolution.

3. Change in pore size distribution

Each pore size of rock samples was extracted and characterized by CT scanning to quantitatively analyze the pore size characteristics of rock samples, and the average pore size of carbonate samples before and after dissolution under different test conditions was obtained. The average pore diameter increased by 0.027 mm, 0.013 mm, 0.013 mm, and 0.034 mm from 0.075 mm, 0.077 mm, 0.065 mm, and 0.082 mm before dissolution, resulting in a 1.36, 1.17, 1.2, and 1.41-fold increase in the initial state of the average aperture ratio.

**Figure 10:**
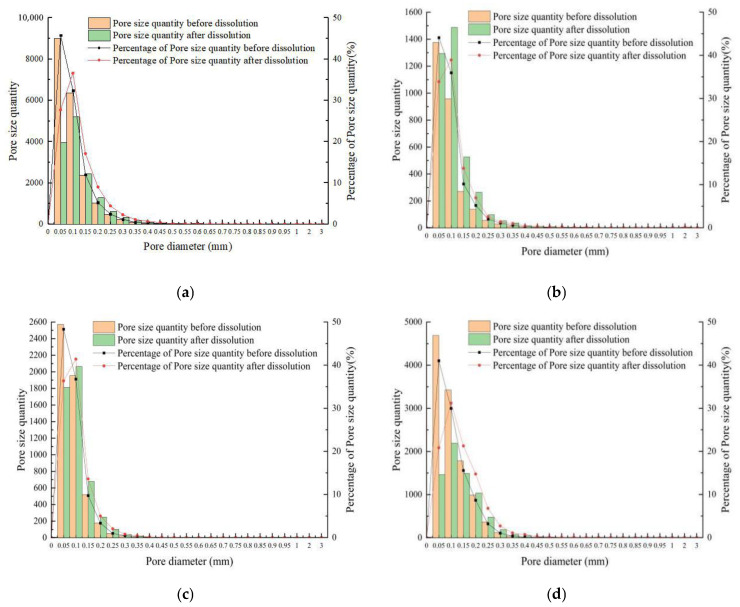
Relationship between pore number and the corresponding pore percentage for different pore diameters: (**a**) sample 5#; (**b**) sample 6#; (**c**) sample 7#; (**d**) sample 8#.

To thoroughly examine the pore size characteristics of the four groups of rock samples, the pore number and pore size percentage were statistically classified from the angle of a single pore diameter after the overall description of the average. Figure 10 shows the pore size distribution (PSD) before and after dissolution. Before dissolution, the PSD of carbonate rocks in four test conditions was logarithmic right, with many pore diameters less than 0.05 mm and a pore size percentage greater than 40%. Regardless of the changes in test conditions after dissolution, the PSD remained right but shifted slightly to the left, indicating that the pore diameter increased. When the pore diameter was greater than 0.15 mm, the number after the dissolution was above that before dissolution. The PSD curve was flatter than before dissolution, indicating a decrease in the number. The increased PSD skewness after dissolution confirmed the increase in the overall aperture. Furthermore, the reduced PSD kurtosis indicated increased aperture ratios scattered around the median value before dissolution.

Compared with the change law of dissolution results, there was no direct correlation between pore size change and dissolution rate. Hence, heterogeneity and numerous unstable carbonate minerals were the key factors for the pore size increase.

### 3.3. Analysis of NMR Test Results

T_2_ spectrum analysis was conducted on the rock samples (5#, 6#, 7#, and 8#), and NMR test results were obtained, including T_2_ spectrum curve, total atlas area, number of wave peaks, and corresponding peak area. The starting and ending points of peaks were also acquired.

#### 3.3.1. T_2_ Spectrum Analysis

The T_2_ distribution is related to pore size, while the number and size of each sample peak value can be clearly and intuitively seen. The T_2_ spectrum ranging from small to large represents micropores, small pores, mesoporous pores, large pores, and cracks, respectively. According to research results [66], the three spectral peaks corresponding to the T_2_ spectrum of 0.5–2.5 ms, 20–50 ms, and greater than 100 ms, represent adsorption pore, seepage pore, and fracture, respectively, for physical property analysis. The larger the spectral peak, the more developed the pore fissure.

**Figure 11:**
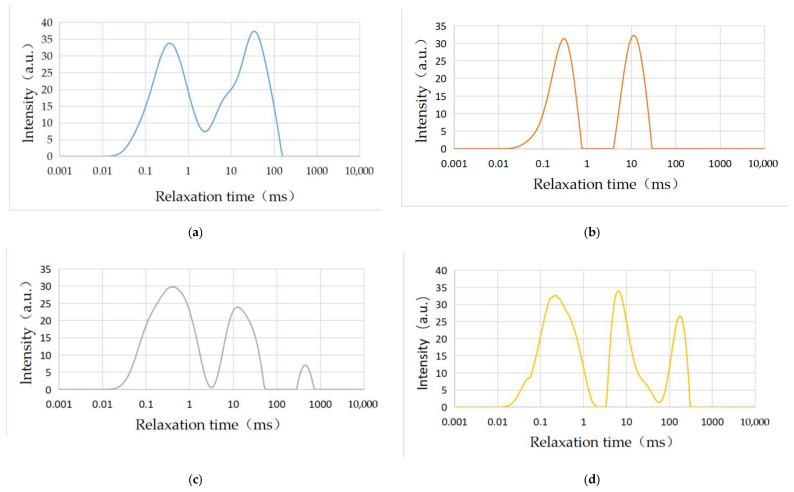
T_2_ spectrum of carbonate samples after dissolution under different test conditions: (**a**) sample 5#; (**b**) sample 6#; (**c**) sample 7#; (**d**) sample 8#.

Figure 11 shows the T_2_ spectrum of four groups of rock samples. It is obvious that two peaks appear in rock samples 5# and 6#, while three peaks appear in rock samples 7# and 8#. The first and second peak wave of sample 5# appeared at 0.005 ms and 2.582 ms, while the end time was 2.382 ms and 148.202 ms, respectively. Additionally, the adsorption space and seepage space developed while connectivity between the two types of pores was good. Meanwhile, the adsorption capacity and seepage conditions were strong. The first and second peak wave of sample 6# appeared at 0.005 ms and 4.199 ms, while the end time was 0.766 ms and 27.050 ms, respectively. In addition, the adsorption space and seepage space developed while connectivity between the two types of pores was weak. Meanwhile, the adsorption capacity and seepage conditions were strong. The first, second, and third peak waves of rock sample 7# appeared at 0.005 ms, 3.293 ms, and 283.309 ms, while the end time was 3.037 ms, 57.709 ms, and 690.551 ms, respectively. Meanwhile, the adsorption space and seepage space developed with a few crack spaces. Additionally, connectivity between the three types of pores was weak while the adsorption capacity and seepage conditions were strong. The first, second, and third peak wave of rock sample 8# appeared at 0.005 ms, 3.571 ms, and 60.802 ms, while the end time was 1.868 ms, 56.072 ms, and 307.211 ms, respectively. Meanwhile, the adsorption space and seepage space developed with a small part of fracture space. In addition, connectivity between the three types of pores was weak while the seepage condition was strong.

#### 3.3.2. Pore Structure Analysis

Table 8 shows the NMR test results of four rock samples, including the time point of wave crest, the area of each peak, and the peak proportion.

The total spectrum area can quantitatively show the difference of total porosity between different samples, which is consistent with the porosity distribution extracted by CT analysis. The value of rock sample 5# is the largest and that of rock sample 6# is the smallest.

**Figure 12:**
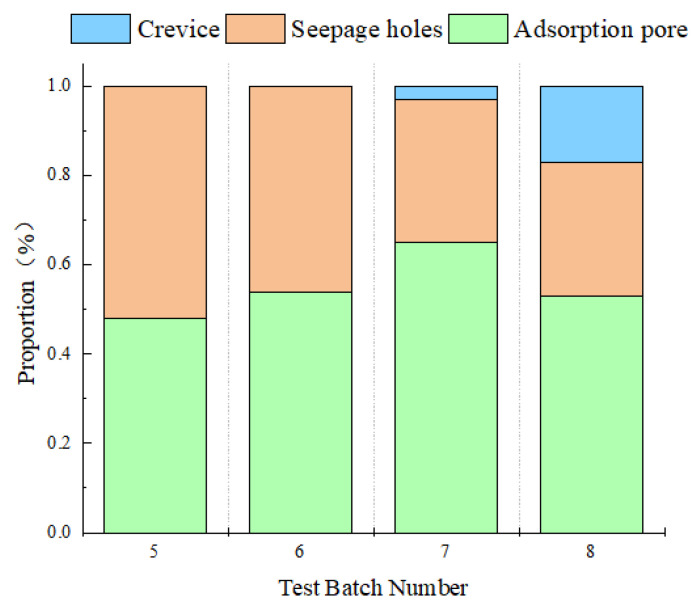
Proportion of the aperture distribution after dissolution under different test conditions.

Figure 12 can clearly and directly show the pore diameter proportion of each sample which is mainly composed of adsorption pore, seepage pore, and fracture. The distribution pattern of different rock pore sizes is essentially consistent with that obtained by CT scanning. After dissolution, pore diameter increases, and dispersed pore sizes near the median value increase.

## 4. Summary and Conclusions

In this study, self-developed carbonate dissolution test equipment was used to perform dynamic dissolution and CT tests on carbonate rocks under thermal–hydraulic–chemical coupling conditions and to investigate the dissolution effect and micro-development law of carbonate rocks under complex conditions. The main conclusions are as follows:

1. The highest dissolution rate can be achieved under working condition 4 (when the five influence factors of the test are set at pH 4.6, 8 MPa, 85 °C, 75 mL·min^−1^, and 21 days). The rate is 364.43 mg/g. The lowest dissolution rate can be attained under working condition 16 (when five influence factors of the test are set at pH 7.0, 8 MPa, 25 °C, 55 mL·min^−1^, and 7 days). The lowest rate is 3.69 mg/g. The dissolution rate is proportional to the flow velocity, temperature, and hydrodynamic pressure and inversely proportional to the pH. Furthermore, the sensitivity, significance, and contribution rate of each influencing factor to each parameter were determined using range analysis and variance analysis. The most influential factor in the dissolution effect is pH, followed by time, temperature, current speed, and hydrodynamic pressure. Moreover, pH has the greatest contribution rate to the erosion rate of rock samples, accounting for 42.36%, followed by time, temperature, current speed, and hydrodynamic pressure.
2. Carbonate rocks have a complex pore space system with different pore compositions at the same facilities. The initial porosity of the four rock samples (5#–8#) was 0.189%, 0.023%, 0.029%, and 0.115%, and an increase of 0.140%, 0.038%, 0.042%, and 0.126%, respectively, was observed after the dissolution. The dissolution can improve overall porosity, and the initial porosity of carbonate rock significantly affected the increase in the dissolution-induced porosity. The change in porosity runs throughout the entire sample; the porosity of the upper part is greater than that of the lower part [67].
3. The total number of pores decreases, while the volume of pores increases after rock dissolution. The number and percentage distribution of pores before and after dissolution increase and decrease as the single pore volume increases [68]. The percentage distribution of pore volume before dissolution has the characteristics of right deviation. Moreover, the percentage distribution of pore volume after dissolution has the characteristics of left deviation, and the peak distribution is flatter than before dissolution. When the single pore volume exceeds 0.0001 mm^3^, the pore volume percentage significantly increases. After dissolution, the average pore size of rock samples increased with the same distribution trend. The corresponding pore size decreased as the single pore size increased before and after dissolution. The PSD of carbonate rocks is logarithmic right deviation before dissolution and slightly left deviation after dissolution. The increased PSD skewness confirms the increase in overall pore size, and the decreased PSD kurtosis shows more aperture ratios scattered around the median before dissolution [69].

The dissolution of carbonate rocks is controlled by karst thermal–hydro–chemical factors, followed by groundwater pH value, reaction time, temperature, groundwater runoff change, and hydrodynamic pressure. The micropore structure can regulate the quality of dissolved pore formation. Furthermore, rock salt is an uneven medium with initial damage, primarily micropores and microcracks. When acid fluid migrates and reacts in the internal pores of rocks, minerals on the pores’ edges dissolve, causing changes in the pore space. Furthermore, some pores merge to form larger pores during the dissolution process. As pore volume increases, the number and percentage of pores increase and decrease. The altered pores alter the fluid migration process in the rock, exacerbating the formation of dissolution pores. Moreover, most dissolved pores are the result of initial pore enlargement rather than the formation of a new pore system. Heterogeneity, the presence of unstable minerals, and a large initial pore size result in the formation of large pores and a new pore system, which are also key factors in pore size increase. This study provides a foundation for predicting the dissolution effect and the evolution law of dissolved pores in carbonate rocks under multifactor coupling conditions. It has important guiding implications for engineering design and construction in karst areas.

Different mesostructure evaluation methods have their respective advantages and disadvantages. Pore structure characteristics of rock samples are observed using CT, and are roughly consistent with the change of carbonate rock porosity in NMR. The characterization of the overall distribution of pores is also consistent [34,70,71]. The porosity of the rock sample is larger than that before corrosion, and corrosion preferentially affects the large pore size. However, the CT scan field of view is too small and limited by the number of rock samples [72]. For the further identification of carbonate rocks, the NMR experiment can be combined with the CT scanning experiment technology. The DWI method makes it possible to study 3D fluid flow, directly understand the connectivity of pore space, and contribute dynamic value to standard imaging techniques [37]. Additionally, by taking μCT technology as reference visualization technology, a fine description of the scale and spatial development and distribution characteristics of pores and fissures should be conducted, to establish the NMR identification method of core pores and fractures in carbonate reservoirs to obtain more perfect pore structure data and provide assurance for the safety evaluation of karst geological disasters [73,74].

## Figures and Tables

**Table 1:** XRD analyses of carbonate rocks [7].

Mineral Composition	Quartz	Calcite	Else
Mineral content (%)	1.5	96.5	2.0

Note: Other substances mainly include weak crystallization or amorphous substances.

**Table 2:** Main chemical composition content of the rock sample and LS (wt%) [7].

Chemical Components	$SiO_{2}$	CaO	$Fe_{2}O_{3}$	$CO_{2}$	MgO	$Al_{2}O_{3}$	SrO	Cl	Loss on Ignition
Mineral content (%)	0.532	55.41	0.206	43.54	0.050	0.053	0.052	0.040	0.117

**Table 3:** Research level of orthogonal numerical simulation experimental parameters.

Level	Dissolution Conditions				
	pH Value	Pressure (MPa)	Temperature (°C)	Current Speed (mL·min^−1^)	Duration (d)
1	4.6	2	25	15	3
2	5.4	4	45	35	7
3	6.2	6	65	55	14
4	7.0	8	85	75	21

**Table 4:** Orthogonal numerical simulation experimental schemes.

Experimental Scheme	Dissolution Conditions Parameters				
	pH Value	Pressure (MPa)	Temperature (°C)	Current Speed (mL·min^−1^)	Duration (d)
1	4.6	2	25	15	3
2	4.6	4	45	35	7
3	4.6	6	65	55	14
4	4.6	8	85	75	21
5	5.4	2	45	55	21
6	5.4	4	25	75	14
7	5.4	6	85	15	7
8	5.4	8	65	35	3
9	6.2	2	65	75	7
10	6.2	4	85	55	3
11	6.2	6	25	35	21
12	6.2	8	45	15	14
13	7.0	2	85	35	14
14	7.0	4	65	15	21
15	7.0	6	45	75	3
16	7.0	8	25	55	7

**Table 5:** Orthogonal test results.

Test Batch Number	Test Specimen Number	Dissolution Rate (mg·g^−1^)	Test Batch Number	Test Specimen Number	Dissolution Rate (mg·g^−1^)
1	1–1	12.17	9	9–1	24.51
	1–2	14.67		9–2	22.06
	1–3	12.08		9–3	22.00
	1–4	12.22		9–4	22.28
	Average value	12.78		Average value	22.71
2	2–1	65.69	10	10–1	12.25
	2–2	95.12		10–2	12.32
	2–3	65.38		10–3	14.74
	2–4	93.14		10–4	12.29
	Average value	79.78		Average value	12.90
3	3–1	155.34	11	11–1	19.75
	3–2	200.00		11–2	24.45
	3–3	141.49		11–3	22.00
	2–4	202.93		11–4	22.06
	Average value	174.76		Average value	22.07
4	4–1	344.66	12	12–1	22.17
	4–2	373.17		12–2	24.51
	4–3	373.13		12–3	24.57
	4–4	366.75		12–4	24.57
	Average value	364.43		Average value	23.96
5	5–1	108.37	13	13–1	9.85
	5–2	105.65		13–2	14.67
	5–3	105.13		13–3	12.17
	5–4	103.96		13–4	12.17
	Average value	105.78		Average value	12.22
6	6–1	32.02	14	14–1	12.25
	6–2	31.71		14–2	19.85
	6–3	26.57		14–3	17.16
	6–4	34.40		14–4	17.16
	Average value	31.15		Average value	16.59
7	7–1	59.11	15	15–1	9.51
	7–2	59.11		15–2	9.50
	7–3	54.19		15–3	7.82
	7–4	56.23		15–4	9.40
	Average value	57.16		Average value	9.16
8	8–1	29.27	16	16–1	4.90
	8–2	27.30		16–2	2.46
	8–3	22.11		16–3	4.90
	8–4	26.89		16–4	2.47
	Average value	26.40		Average value	3.69

**Table 6:** Extreme range analysis of dissolution rate of specimens (mg·g^−1^).

Level	pH Value	Pressure (MPa)	Temperature (°C)	Current Speed (mL·min^−1^)	Duration (d)
1	157.938	38.373	17.422	27.623	15.310
2	55.123	35.105	54.670	35.117	40.835
3	20.410	65.787	60.115	74.282	60.523
4	10.415	104.620	111.678	106.862	127.218
Range	147.523	69.515	94.256	79.239	111.908

**Table 7:** Variance analysis of dissolution rate of the specimen (mg·g^−1^).

Factor	Deviation Quadratic Sum	Degree of Freedom	F Comparison	F Critical Value	Contribution Rate (%)
pH Value	54,551.244	3	2.118	3.290	42.36
Pressure (MPa)	12,432.704	3	0.483	3.290	9.65
Temperature (°C)	18,032.225	3	0.700	3.290	14.00
Current Speed (mL·min^−1^)	16,255.007	3	0.631	3.290	12.62
Duration (d)	27,516.741	3	1.068	3.290	21.37
Deviation	128,787.92	15	-	-	-

**Table 8:** Magnetic resonance data of carbonate rocks.

Rock Sample	Total Spectral Area	Wave Peaks	Peak Point Time	Peak Area	Peak Proportion (%)
5#	2106.97	1	0.369	1012.26	0.48
		2	34.489	1094.71	0.52
6#	1075.75	1	0.289	583.43	0.54
		2	11.098	492.32	0.56
7#	1601.11	1	0.289	1034.44	0.65
		2	12.034	512.41	0.32
		3	460.592	53.66	0.03
8#	1792.93	1	0.227	946.82	0.53
		2	6.295	546.39	0.30
		3	174.263	299.72	0.17

## Data Availability

The data used to support the findings of this study are available from the corresponding author upon request.
